# Advanced strategies to evade the mononuclear phagocyte system clearance of nanomaterials

**DOI:** 10.1002/EXP.20220045

**Published:** 2023-01-05

**Authors:** Junjie Lu, Xiao Gao, Siyao Wang, Yuan He, Xiaowei Ma, Tingbin Zhang, Xiaoli Liu

**Affiliations:** ^1^ Key Laboratory of Synthetic and Natural Functional Molecule of the Ministry of Education College of Chemistry and Materials Science Northwest University Xi'an China; ^2^ Key Laboratory of Resource Biology and Biotechnology in Western China of the Ministry of Education School of Medicine Northwest University Xi'an China; ^3^ Institute of Regenerative and Reconstructive Medicine Med‐X Institute National Local Joint Engineering Research Center for Precision Surgery & Regenerative Medicine Shaanxi Provincial Center for Regenerative Medicine and Surgical Engineering First Affiliated Hospital of Xi'an Jiaotong University Xi'an China; ^4^ National Center for Veterinary Drug Safety Evaluation College of Veterinary Medicine China Agricultural University Beijing China

**Keywords:** biomimetic nanotechnology, drug delivery, macrophage blockade, mononuclear phagocytic system, nanomaterials, surface modification

## Abstract

Nanomaterials are promising carriers to improve the bioavailability and therapeutic efficiency of drugs by providing preferential drug accumulation at their sites of action, but their delivery efficacy is severely limited by a series of biological barriers, especially the mononuclear phagocytic system (MPS)—the first and major barrier encountered by systemically administered nanomaterials. Herein, the current strategies for evading the MPS clearance of nanomaterials are summarized. First, engineering nanomaterials methods including surface modification, cell hitchhiking, and physiological environment modulation to reduce the MPS clearance are explored. Second, MPS disabling methods including MPS blockade, suppression of macrophage phagocytosis, and macrophages depletion are examined. Last, challenges and opportunities in this field are further discussed.

## Graphical abstract

Mphagocyticononuclear system (MPS) is one of the major obstacles that impede the accumulation of nanomaterials at their target sites. This Review summarizes the advanced strategies for evading the MPS clearance of nanomaterials, and discusses the challenges and opportunities in this field.

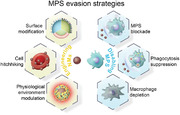

## 1 INTRODUCTION

Nanomaterials (NMs) with unique physicochemical properties such as tunable size, high surface area‐to‐volume ratio, abundant chemical composition, and easily functionalized surface are promising carriers to transport drugs to target sites.^[^ ^1^ ^]^ So far, various NMs with different functions have been developed to increase the therapeutic efficacy and decrease harmful side effects of drugs.^[^ ^2^ ^]^ However, although many nanocarriers have been reported to show favorable effect in vitro, when applied in vivo, poor bioavailability is often observed, and the administered NMs are prone to massively accumulate in off‐target tissues. For example, Wilhelm et al. analyzed the tumor delivery efficiency of NMs reported from 2006 to 2016, and found that only 0.7% (median) of the administered NMs were successfully delivered to solid tumors.^[^ ^3^ ^]^ The poor delivery efficiency of NMs is largely due to sequential biological barriers that prevent successful accumulation of NMs in targeted sites, such as MPS sequestration, blood vessel extravasation, cellular internalization, and endosomal escape.^[^ ^4^ ^]^

**FIGURE 1:**
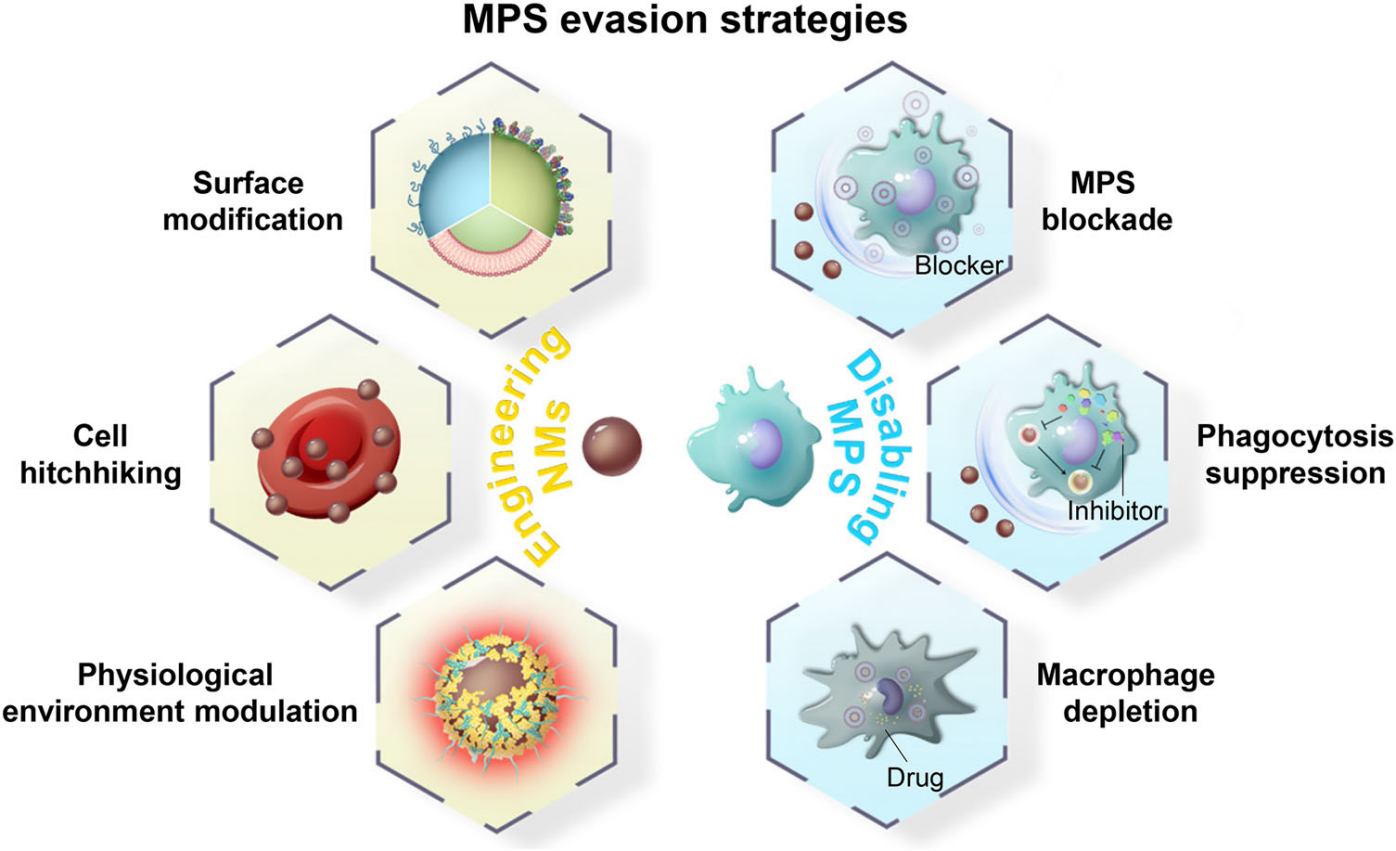
Schematic showing the strategies for circumventing the clearance of NMs by the MPS.

MPS is the first and major obstacle blocking the systemic administration of NMs to target sites, and most of the injected doses are already lost after MPS clearance.^[^ ^5^ ^]^ The MPS consists of a network of phagocytes, including macrophages, monocytes, and dendritic cells, in all organs, especially the liver, spleen, and lymph nodes that contain resident macrophages.^[^ ^6^ ^]^ The process of sequestration by MPS begins with opsonization of NMs. During this process, adsorption of opsonins such as complement proteins, immunoglobulins, and fibrinogen on the surface of circulated NMs occurred, and then NMs are internalized by macrophages.^[^ ^7^ ^]^ In recent years, various approaches have been developed to delay, reduce, or even entirely avoid the elimination of NMs by the MPS. In this review, we summarized and divided these approaches into two categories, as shown in Figure 1. The first strategy is to engineer NMs, including surface modification (surface coating using polymers, proteins, and cell membranes), cell hitchhiking and physiological environment modulation, to prevent their interactions with phagocytic cells of the MPS. Approaches on changing the sizes, shapes, and chemical compositions of NMs to enable the escape of NMs from the MPS sequestration have been reviewed extensively elsewhere.^[^ ^8^ ^]^ The second strategy is to disable MPS function to minimize NMs clearance, including MPS blockade, suppression of macrophage phagocytosis, and depletion of macrophages. The present review focuses on recent advances in strategies to evade the MPS clearance of NMs that could improve the accumulation of NMs in the desired sites and achieve improved therapeutic efficacy.

## 2 ENGINEERING NMs FOR STEALTH

There are many possible approaches for engineering NMs to reduce their interactions with MPS cells and thus evade MPS clearance. Herein, we first summarize the surface modifications approaches for NMs to minimize MPS clearance, including coating their surface with polymers, surface pre‐adsorption of proteins, and coating cell membranes. Second, we discuss in detail the cell‐hitchhiking approach, including hitchhiking of NMs NMon red blood cells (RBCs), neutrophils, monocytes/macrophages, lymphocytes, and stem cells. Last, we introduce the approach via changing the external physiological environments of NMs to facilitate their escape from MPS.

### 2.1 Surface modifications of NMs

#### 2.1.1 Surface coating using polymers

Surface coating of NMs with polymers is typically used to facilitate NMs to escape from the MPS clearance. In this method, the grafted polymers will form a physical barrier on the surface of NMs to resist non‐specific absorption of serum proteins.^[^ ^9^ ^]^ For example, poly(ethylene glycol) (PEG) is one of the best‐known surface coating polymer.^[^ ^10^ ^]^ PEG works by providing a hydrating layer that shields the NMs surface from interacting with opsonins, thus minimizing NMs elimination by the MPS and prolonging their circulation time.^[^ ^11^ ^]^ The in vivo behaviors of PEGylated NMs depend on the molecular weight and surface density of the grafted PEG.^[^ ^12^ ^]^ For instance, elimination of NMs from circulation by the MPS generally decreases with the increase of PEG molecular weight,^[^ ^13^ ^]^ therefore researchers often choose PEGs with molecular weight of 2 kDa or higher.^[^ ^14^ ^]^ In terms of the surface density of PEG, it is generally believed that a highly dense coating of PEG adopts a “brush” or “dense brush” conformation, and this conformation reduces the recognition of NMs by phagocytic cells.^[^ ^15^ ^]^ However, PEGylation alone does not completely protect NMs from recognition by MPS, as it cannot fully shield NMs surfaces from serum protein adsorption.^[^ ^16^ ^]^ Furthermore, repeated administration of PEGylated NMs can result in induction of anti‐PEG antibodies in both of animal models and patients. High concentrations of such antibodies would lead to even more rapid NMs clearance, which is called the accelerated blood clearance (ABC) phenomenon.^[^ ^17^ ^]^

Zwitterionic polymers comprise another notable class of antifouling polymers. They consist of equal numbers of cationic and anionic groups, thereby showing an overall neutral charge.^[^ ^18^ ^]^ This characteristic enables the zwitterionic polymers to form a hydration layer via electrostatic interactions that is more tightly bound than PEG modification that is maintained by hydrogen bonds. The antifouling capacity of zwitterionic polymers is equivalent, or even superior, to that of PEG. Moreover, these polymers do not induce ABC phenomenon.^[^ ^19^ ^]^ Furthermore, unlike PEG, which consists of the same repeating unit, zwitterionic polymers have a broader chemical diversity, due to their abundant cationic and anionic moieties, as well as the spatial arrangement of their charged moieties.^[^ ^20^ ^]^ In the biomedical field, the most widely used zwitterionic polymers are poly(sulfobetaine) (PSB), poly(carboxybetaine) (PCB), and poly(phosphorylcholine) (PPC).^[^ ^21^ ^]^ Recently, Peng et al. showed that Fe_3_O_4_ NMs coated with zwitterionic polymers, such as PSB, PCB, and PPC, could resist protein adsorption, and achieved longer blood circulation times than those RBC membrane‐coated Fe_3_O_4_.^[^ ^22^ ^]^ Zwitterionic polypeptide is another type of zwitterionic polymer, an emerging antifouling coating candidate, which is known to be biodegradable and monodisperse, and it is possible to precisely control the site and stoichiometry of its functional moieties.^[^ ^23^ ^]^

**FIGURE 2:**
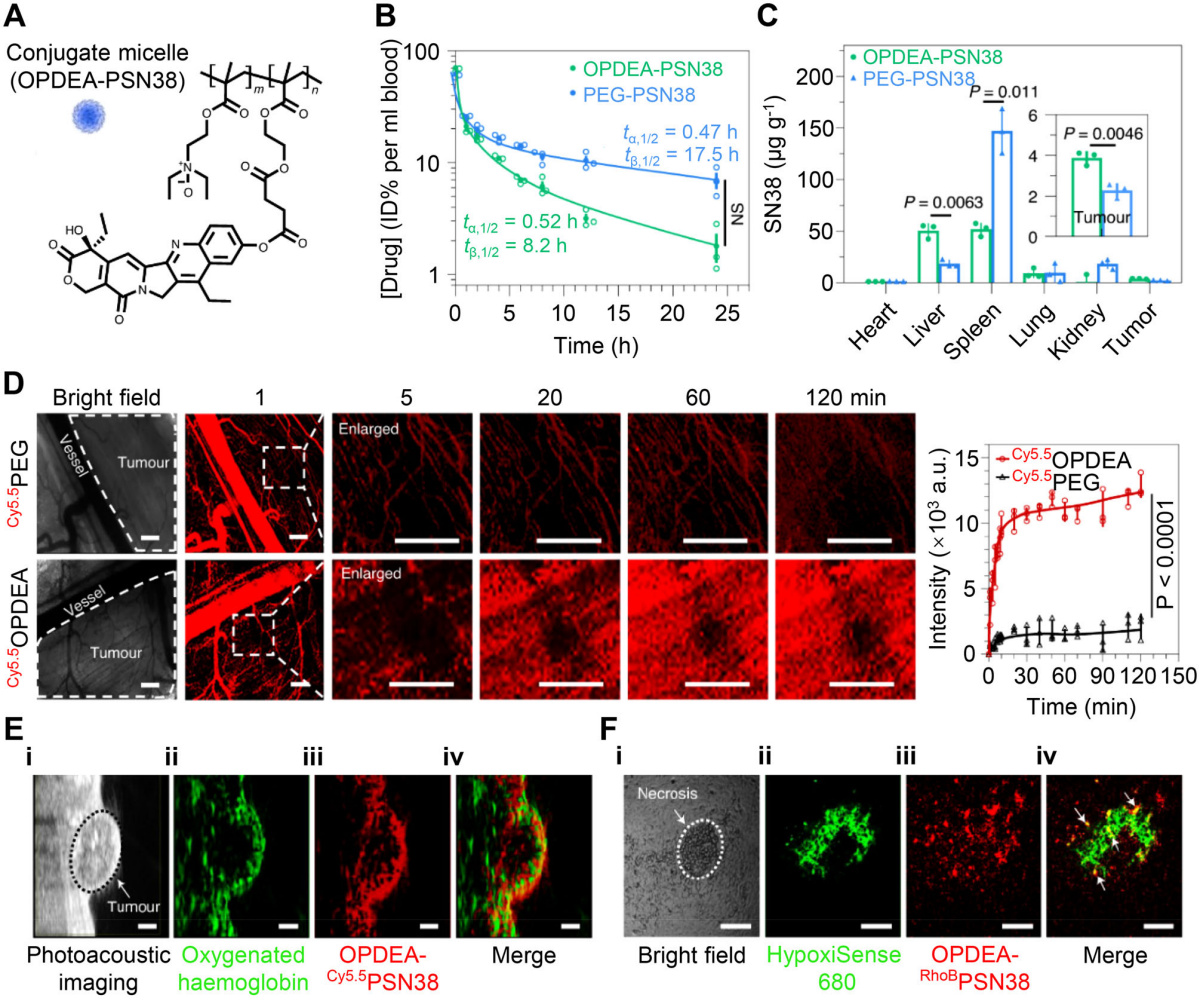
Zwitterionic OPDEA‐PSN38‐based micelle for efficient cancer drug delivery. (A) Molecular structure of OPDEA‐PSN38, which can form micelles in aqueous solution. Blood clearance (B) and biodistribution (C) of OPDEA‐PSN38 and PEG‐PSN38 micelles in HepG2 tumor‐bearing mice. The content of SN38 was analyzed 8 h after intravenous injection (SN38‐equivalent dose, 10 mg kg^−1^). (D) In vivo fluorescence imaging showing the extravasation of OPDEA or PEG from tumor blood vessels into tumor tissue in a subcutaneous 4T1 tumor after intravenous administration. Both polymers were labeled with Cy5.5 (red) (Cy5.5‐equivalent dose, 0.5 mg kg^−1^). The left pictures show representative and enlarged images of a randomly selected location (white dashed frame), and the right panel shows the overall fluorescence intensity of the selected area. Scale bars, 500 μm. (E) In vivo ultrasound and photoacoustic images showing the distribution of OPDEA‐^Cy5.5^PSN38 micelles inside a HepG2 tumor (Cy5.5‐equivalent dose, 0.5 mg kg^−1^). (i) Ultrasound image of the tumor; (ii) photoacoustic image of the oxygenated hemoglobin used to outline the hypoxic area in the tumor (green); (iii) fluorescent image of the micelles (red) in the tumor; (iv) overlapped image of oxygenated hemoglobin and the micelles. Scale bars, 1.0 mm. (F) Ex vivo CLSM images showing the distribution of circulated RhoB‐labeled OPDEA‐PSN38 micelles in hypoxic tumor regions at 6 h. The tumor received a RhoB‐equivalent dose of 0.5 mg kg^−1^. The hypoxic regions in a tumor were stained with HypoxiSense 680. (i) A necrotic region marked by a white dashed line; (ii) HypoxiSense 680 (green); (iii) OPDEA‐^RhoB^PSN38 (red); (iv) overlapped image, white arrows indicate colocalization (yellow dots). Scale bars, 100 μm. Reproduced with permission.^[^ ^27^ ^]^ Copyright 2021, Springer Nature.

Zwitterionic polymers have also been used to construct non‐immunogenic NMs, such as micelles,^[^ ^24^ ^]^ liposomes,^[^ ^25^ ^]^ and hydrogels.^[^ ^26^ ^]^ For example, a drug‐conjugate micelle was developed by Chen et al. for cancer drug delivery by taking advantage of the self‐assembly of the amphiphilic block copolymers, in which the zwitterionic poly(2‐(*N*‐oxide‐*N*,*N*‐diethylamino)ethyl methacrylate) (OPDEA) served as the hydrophilic block and the polymer containing 7‐ethyl‐10‐hydroxycamptothecin (SN38) served as a hydrophobic block (Figure 2A).^[^ ^27^ ^]^ The resulting OPDEA‐based micelle did not stick to serum proteins while binding reversibly to cell membranes via the interactions between the *N*‐oxide moiety of OPDEA and the phosphatidylcholine (PC) or phosphatidylethanolamine (PE) in the cell membrane. These features allowed the micelle to circulate longer in the blood (elimination half‐life, 8.2 h), accumulate efficiently in tumors (twice higher than PEG‐PSN38 micelle), and penetrate deeply into the tumors via transcytosis‐based intercellular delivery (Figure 2B–F). Peng et al. developed a hyperthermia‐responsive zwitterionic nanogel for cancer drug delivery using sulfamide‐based zwitterionic moieties as the monomer and bis(acryloyl)cystamine (BAC) and ethyleneglycol dimethacrylate (EGDMA) as the crosslinker.^[^ ^28^ ^]^ The zwitterionic nanogel showed prolonged circulation, 2.95‐fold higher than PEGylated nanogel. Furthermore, the nanogel could collapse at 37°C and change to the swelled state after microwave heating. Therefore, upon transferrin modification, microwave‐triggered tumor accumulation and drug release could be achieved.

In addition to antifouling zwitterionic polymers, functional zwitterionic materials have been developed to render NMs with additional functionalities, such as stimuli‐sensitivity, crossing blood‐brain barrier (BBB), imaging and immunomodulation, etc.^[^ ^29^ ^]^ Li et al. designed a functional zwitterionic phosphoserine‐mimetic polymer (ZPS) built from phosphoserine, an immunomodulatory molecule.^[^ ^29b^ ^]^ The produced ZPS can actively suppress undesired immune system activation while showing decreased vulnerability to phagocyte clearance. ZPS represents the first zwitterionic polymer that simultaneously exhibits immunomodulatory and non‐fouling functions.

#### 2.1.2 Surface pre‐adsorption of proteins

Surface pre‐adsorption of proteins on NMs has also been studied as a possible approach to achieve MPS escape.^[^ ^30^ ^]^ Dysopsonic proteins such as human serum albumin (HSA) and apolipoprotein E (ApoE) are commonly used for non‐covalent adsorption on NMs prior to administration.^[^ ^31^ ^]^ The pre‐coated proteins can prevent the adsorption of plasma opsonins through steric blocking and thus confer on NMs stealth properties to evade MPS sequestration.^[^ ^32^ ^]^ Although the pre‐coated proteins may be partially exchanged or covered by serum proteins when injected in vivo, this approach is shown to be effective in several experiments. For example, Lu et al. demonstrated that ApoE pre‐coating substantially decreased the accumulation of graphene in MPS‐related tissues such as those in spleen and liver as compared with pristine graphene.^[^ ^33^ ^]^ Furthermore, ApoE pre‐coating also markedly extended the circulation of gold nanoparticles (AuNPs) in blood. Park et al. showed that pre‐adsorption of proteins such as bovine serum albumin, HSA, complement factor H, and fibrinogen on silica nanoparticles not only diminished macrophage uptake but also inhibited nanoparticle‐induced complement activation.^[^ ^34^ ^]^

In addition to reducing the MPS sequestration, the pre‐coated proteins can also endow NMs with additional functionalities such as active targeting capability. Tonigold et al. showed that the pre‐coated antibodies on NMs remained functional even in 100% serum, whereas the antibodies covalently coupled to NMs lost nearly all their ability to bind to antigen under the same conditions.^[^ ^35^ ^]^ Oh et al. used a recombinant fusion protein composed of a combination of HER2‐binding affibody with glutathione‐*S*‐transferase to pre‐coat mesoporous silica nanoparticles (MSNs).^[^ ^32^ ^]^ The supramolecular pre‐coated proteins significantly decreased the adsorption of opsonins such as complement proteins and immunoglobulins on the MSNs as compared with the control MSNs, which enabled the MSNs to escape from the MPS while maintaining their targeting abilities. As a result, the accumulation of the protein‐coated MSNs in tumors was 2.5‐fold greater than that of the control MSNs.

#### 2.1.3 Surface coating using cell membranes

Cell membrane‐coating is a biomimetic nanotechnology that integrates the positive attributes of natural and synthetic systems. It is a promising approach to evade MPS clearance of NMs, owing to the natural function originating from cell membranes, such as immune evasion, long circulation, disease‐relevant targeting, etc.^[^ ^36^ ^]^ This approach was first demonstrated by Zhang and coauthors in 2011, in which they showed that RBC membrane‐coating greatly extended the circulation time of PEGylated NMs from 15.8 to 39.6 h.^[^ ^37^ ^]^ Afterward, the cell membrane‐coating nanotechnology is expanded to various types of cells, such as platelets, macrophages, neutrophils, cancer cells, and many others (Table 1).^[^ ^38^ ^]^ The successful application of this nanotechnology depends on not only effective methods for collecting, purifying, and coating membranes, but also the biological function of the source cell membranes.

**TABLE 1:** Coating methods and key features of biological membranes

Biomembrane types	Coating methods	Key features	Ref.
RBC membrane	Physical extrusion, sonication, microfluidic devices	Easily collected, immune evasion, long circulation time	[40, 41, 42, 43]
Platelet membrane	Physical extrusion, sonication, sonication + extrusion	Immune evasion, long circulation time, targeting injured blood vessels, pathogen adhesion, targeting tumor cells^a^	[45, 46, 47, 48]
Leukocyte membrane	Physical extrusion, sonication, sonication + extrusion	Immune evasion, inflammatory targeting, actively across BBB^b^	[50, 51, 52, 53, 54]
Cancer cell membrane	Physical extrusion, sonication	Homologous targeting properties, stimulating tumor‐specific immunity, easily obtained	[55, 56, 57]
Stem cell membrane	Physical extrusion, sonication	Immune evasion, intrinsic tropism to inflammation, and multiple malignancies^c^	[59, 65]
Bacteria membrane	Physical extrusion, sonication	Stimulating innate immunity, effective internalization by leukocytes	[58, 60]
Hybrid cell membrane	Physical extrusion, sonication, sonication + extrusion	Multifunctional integration of different cells	[61, 62, 63, 64]

^a^Targeting the tumor cells that express CD41, CD61 and P‐selection receptors.

^b^Macrophage or neutrophil membrane‐coated NMs are able to actively across the BBB.

^c^Targeting tumors such as breast cancer, colon cancer, glioma, and hepatocellular carcinoma.

RBCs are the most well‐studied cell type for constructing cytomembrane‐cloaked NMs.^[^ ^39^ ^]^ On one hand, they are the most abundant cell type, and mature RBCs have no nuclei and complex organelles. On the other hand, these cells express multiple surface markers (e.g., self‐marker CD47) that prevent them from clearance by the MPS, resulting in long circulation times (around 120 days). Due to these features, different types of RBC membrane‐coated NMs with extended circulation times have been developed. These NMs include polymer, iron oxide nanoparticles (IONPs),^[^ ^40^ ^]^ upconversion nanoparticles,^[^ ^41^ ^]^ AuNPs, metal‐organic framework (MOF),^[^ ^42^ ^]^ etc., which are made via extrusion, sonication, or microfluidic devices.^[^ ^43^ ^]^ In a more recent study, Ben‐Akiva et al. showed that anisotropic shapes (e.g., prolate ellipsoidal and oblate ellipsoidal) of polymeric NMs can act in synergy with the RBC membrane‐coating to evade elimination by the MPS and achieve longer circulation times.^[^ ^44^ ^]^

Platelets are small, circulating anucleate cells that are another important source of membranes.^[^ ^45^ ^]^ The platelet membrane can easily be obtained by freezing and thawing platelets repeatedly. The resulting membrane is then coated onto the NMs cores. Platelet membrane cloaking has been shown to evade sequestration by the MPS and endow the NMs with new functionalities such as selective binding to injured vessels, pathogen adhesion, and targeting tumor cells with overexpressed surface markers such as CD61, CD41, and P‐selection receptors.^[^ ^46^ ^]^ In one example, a platelet membrane‐coated MOF system effectively delivered siRNA to the SK‐BR‐3 breast tumors that express CD24, a counter receptor to P‐selectin. Notably, the platelet membrane‐coated MOF achieved a 6‐fold greater accumulation in tumors compared with the RBC membrane‐coated MOF.^[^ ^47^ ^]^ Rao et al. showed that photothermal therapy (PTT)‐mediated damage to tumor vessels could further improve the accumulation of platelet membrane‐coated NMs in MCF‐7 tumors as compared to the untreated controls.^[^ ^48^ ^]^

**FIGURE 3:**
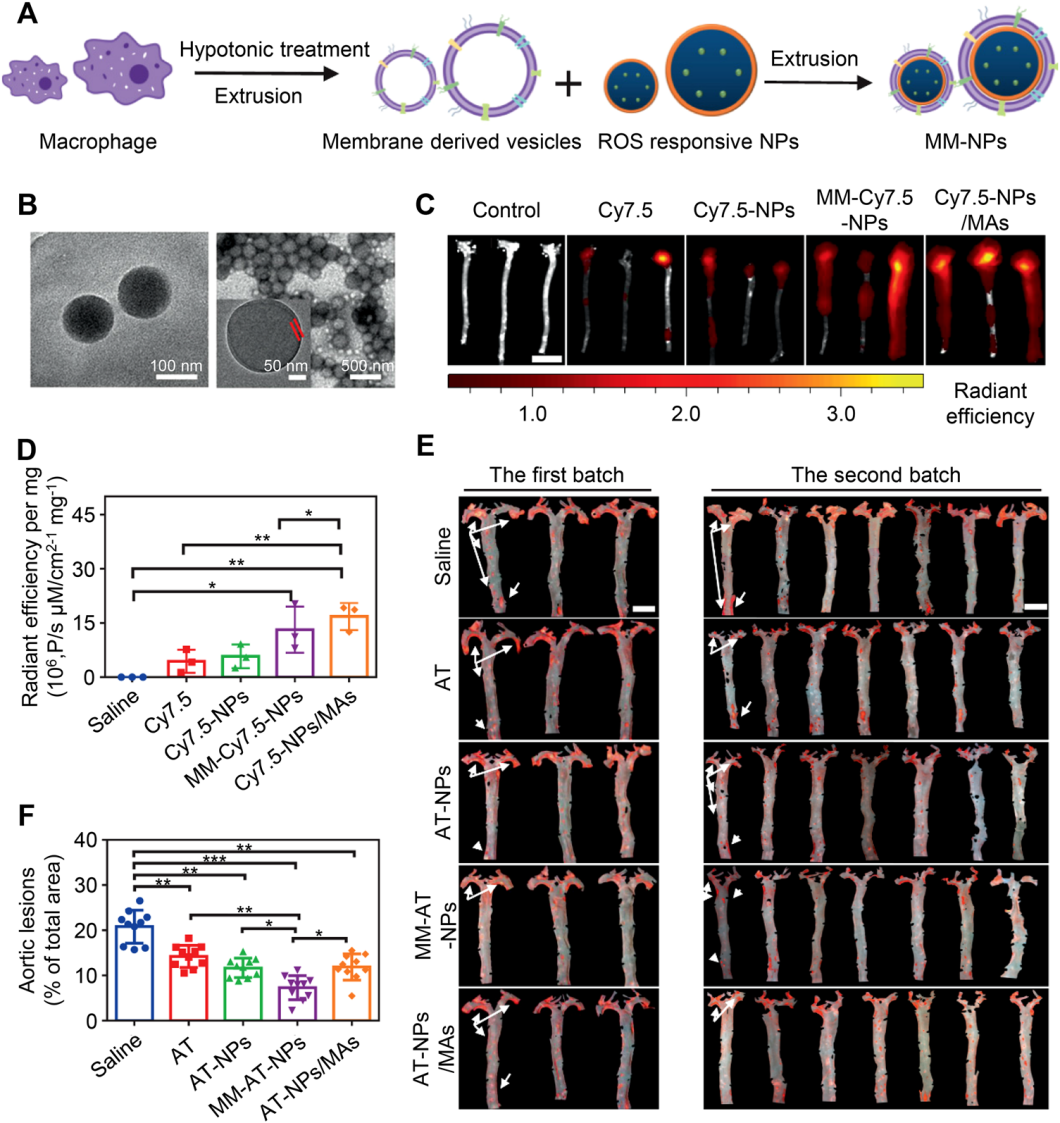
Biomimetic macrophage membrane‐coating nanotechnology for the treatment of atherosclerosis. (A) Schematic diagram of fabrication of the macrophage membrane‐coated NPs (MM‐NPs). (B) TEM images of NPs (left) and MM‐NPs (right). Fluorescence imaging (C) and quantitative analysis (D) of aorta tissues from atherosclerotic mice harvested after injection of different formulations with the same dosage of Cy7.5 at 6 h. Cy7.5 was used to prepare Cy7.5‐loaded NPs (Cy7.5‐NPs), MM‐Cy7.5‐NPs, and Cy7.5‐NPs/MAs. Scale bar, 15 mm. (E) Images of Oil Red O‐stained aorta tissues (red region) in atherosclerotic mice after AT, AT‐NPs, MM‐AT‐NPs, and AT‐NPs/MAs treatments. Scale bars: 5 mm. (F) Quantitative analysis of plaque area relative to total aorta tissue area. Reproduced with permission.^[^ ^51^ ^]^ Copyright 2020, Springer Nature.

Additionally, leukocytes, including macrophages, neutrophils, T cells, and others, that possess immune evasion and excellent inflammatory tropism properties have also been applied to construct the biomimetic NMs.^[^ ^49^ ^]^ Unlike RBCs or platelets, leukocytes are nucleated and have complex intracellular structures. Therefore, it generally requires going through more complicated workflows to separate their intracellular contents from their membranes. In one application of this approach, Yue et al. developed a macrophage membrane‐coated biomimetic polydopamine NMs to remodel the inflammatory microenvironment following PTT.^[^ ^50^ ^]^ The biomimetic NMs were shown to actively target the post‐PTT inflammatory microenvironment, in which the repolarization agent, TMP195, absorbed on polydopamine was released, resulting in the transformation of recruited M2‐like tumor‐associated macrophages to the antitumor M1‐like phenotype. This approach has also been applied to treat atherosclerosis, where the macrophage membrane‐coating not only shielded the atorvastatin‐loaded ROS‐responsive nanoparticles (AT‐NPs) from the MPS and led the NPs to the inflammatory tissues, but also sequestered key proinflammatory cytokines or chemokines that are involved in the atherosclerotic process (Figure 3A–D).^[^ ^51^ ^]^ Additionally, this type of membrane‐coating did not significantly influence the ROS responsiveness of the NPs, thus enabling the release of specific drugs in inflammatory plaques. Compared to free AT‐NPs, the macrophage membrane‐coated AT‐NPs (MM‐AT‐NPs) improved the therapeutics efficacy against atherosclerosis in mice due to the combination of pharmacotherapy and inflammatory cytokines sequestration (Figure 3E,F). In particular, MM‐AT‐NPs showed better therapeutic efficacy than that of AT‐NPs/MAs (AT‐NPs loaded inside living macrophages), though the MA‐based platform exhibited higher accumulation in inflammatory plaques than that of the MM‐based platform (Figure 3C–F). This is probably due to the inherent inflammatory activation of live macrophages. The work suggests that compared to the live macrophage approach, the macrophage membrane‐coating approach is probably more suitable for anti‐inflammatory therapies. Similarly, neutrophil membrane‐coated NMs have been shown to act as an anti‐inflammatory agent for rheumatoid arthritis management.^[^ ^52^ ^]^ Furthermore, because macrophage and neutrophil membrane‐coated NMs are able to actively across the BBB, this approach has been used for treatment of brain diseases such as glioblastoma^[^ ^53^ ^]^ and ischemic stroke.^[^ ^54^ ^]^

In addition to blood cells, cancer cells are also a major source of membranes. These cell membranes have a complete copy of antigenic structure from the source cancer cells, which can endow the NMs with immune evasion and homologous targeting properties and act as an anticancer vaccine.^[^ ^55^ ^]^ For instance, Wang et al. demonstrated that brain metastatic tumor cell membrane‐coating can enable ICG‐loaded polymeric NMs to stay in circulation, across the BBB and accumulate in brain tumor.^[^ ^56^ ^]^ These enabled the encapsulated NMs to achieve superior glioma inhibition by PTT in orthotopic glioma‐bearing mice. Xiong et al. prepared a personalized nanovaccine by cloaking R837‐loaded poly(lactic*‐co‐*glycolic acid (PLGA) NMs with a calcinetin (CRT)‐expressed cancer cell membrane, which was obtained by inducing immunogenic cell death of Luc‐4T1 cells in vitro. The cancer cell membrane antigen array and CRT exposure synergistically increased the active uptake of the nanovaccine by dendritic cells (DCs). In addition, the continuous release of adjuvant R837 from the nanovaccine further activated the DCs, resulting in an improved antitumor immune response.^[^ ^57^ ^]^

Other cell membranes, such as the membranes from the stem cell and bacteria have also been used to coat NMs.^[^ ^58^ ^]^ The stem cell membrane can provide NMs with immune evasion and tumor tropism properties, and thus is appropriate for tumor‐targeted delivery.^[^ ^59^ ^]^ Bacterial membrane‐coating can promote antibacterial immunity and facilitate the uptake of NMs by leukocytes, such as macrophages and neutrophils.^[^ ^60^ ^]^ Furthermore, hybrid cell membranes have been developed from multiple cell types or liposomes to endow the biomimetic NMs with enhanced functionalities.^[^ ^61^ ^]^ For instance, platelet‐leukocyte membrane‐coated immunomagnetic beads were designed with inherent enhanced tumor cell binding capability and reduced interactions with homologous leukocyte. It was found that the beads could efficiently and specifically separate circulating tumor cells from circulation.^[^ ^62^ ^]^ The DC‐tumor fusion membrane‐coated NIR‐II absorbing polymer, having homologous targeting, multicellular engagement, and immune activation abilities, was developed to elicit powerful antitumor immune responses.^[^ ^63^ ^]^ RBC‐retinal endotheliocyte membrane‐coated PLGA NMs with both immune evasion and homotypic targeting capabilities were also developed to achieve noninvasively targeted treatment of choroidal neovascularization.^[^ ^64^ ^]^ Another type of cell membrane‐coating nanotechnology relies on the use of genetically or chemically engineered cell membranes, which offer elaborate means for adding functionalities to NMs, such as cell/virus specific targeting^[^ ^65^ ^]^ and enhanced anticancer immunity.^[^ ^66^ ^]^

### 2.2 Cell hitchhiking

Cell hitchhiking refers to the use of living cells as vehicles to evade the elimination of NMs by MPS. Similar to the cell membrane‐coating approach, cell hitchhiking enables NMs to evade the immune recognition and to seek target tissues via tropism. Moreover, additional functionalities could be achieved by the cell hitchhiking technique. Attributed to the natural phagocytic abilities of neutrophils or macrophages, the natural lung targeting ability of RBCs, and the ability of neutrophils to release payloads in response to inflammatory stimuli, hitchhiking of NMs on these cells will render NMs the corresponding function. However, much attention should be paid when loading NMs to minimize the damage and maintain the physiological function of cells. This approach has been expanded to many types of endogenous cells, including RBCs, neutrophils, monocytes/macrophages, lymphocyte, and stem cells (Table 2).^[^ ^67^ ^]^ In this section,we will discuss key advances in cell hitchhiking approach with a focus on the principal advantages of different cells in NMs delivery.

**TABLE 2:** Selected cell‐hitchhiking platforms

Carrier cells	NMs	Construction methods	Improvement in delivery efficacies^a^	Animal models/targets	Ref.
RBC	IONPs	In vitro surface attachment	120‐fold	Normal mice/lung	[68]
RBC	PS	In vitro surface attachment	7‐fold	Normal mice/lung	[69]
RBC	Liposome	In vitro surface attachment	40‐fold	Normal mice/lung	[70]
RBC	PLGA	In vitro surface attachment	16.6‐fold	Mlanoma lung metastasis mice/lung metastasis	[72]
RBC	PS	In vitro surface attachment	1.5‐fold	Normal mice/spleen	[73]
Neutrophil	Anti‐CD11b‐GNRs	In vivo uptake NMs	20‐fold	Lewis lung carcinoma bearing mice/tumor	[74]
Neutrophil	Liposome	In vitro uptake NMs	N/A	Postsurgical glioma bearing mice/brain tumor	[76]
Neutrophil	MagneticMSNs	In vitro uptake NMs	5‐fold	Postsurgical glioma bearing mice/brain tumor	[77]
Neutrophil, monocyte	cRGD‐liposome	In vivo uptake NMs	N/A	CI/reperfusion rats/ischemic brain	[78]
Neutrophil	Micelle/NPN	In vivo uptake NMs	N/A	Breast cancer bearing mice/tumor	[79]
Macrophage	Liposome	In vitro surface attachment	2.1‐fold	Acute lung inflammation mice/inflamed lung	[81a]
Macrophage	Liposome	In vitro surface attachment	3.02‐fold, 4.1‐fold	Acute pneumonia mice/inflamed lung, melanoma bearing mice/tumor	[83]
Macrophage monocyte	AuNR/ABs	In vivo uptake NMs	N/A	Lymphoma bearing mice/tumor	[86]
Monocyte	c(RGDfc)‐micelle	In vitro surface attachment	N/A	IPF mice/injured lung sites	[81b]
Monocyte	Liposomal quantum dots	In vivo uptake NMs	15‐fold^b^	Breast cancer bearing mice/tumor	[85a]
Monocyte	Aptamer‐liposome	In vivo surface attachment	N/A	Myocardial IR injury mice/IR heart, pancreatic cancer bearing mice/tumor	[87]
T cell	Liposome	In vitro surface attachment	63‐fold	Lymphoma bearing mice/tumor bearing lymph node	[88b]
T cell	DC@AIEdots	In vivo surface attachment	1.6‐fold^c^	Breast cancer bearing mice/tumor	[91]
Stem cell	AuNPs	In vitro uptake NMs	37‐fold	Fibrosarcoma bearing mice/tumor	[93]

^a^The delivery efficacies of cell hitchhiking of NMs relative to free NMs.

^b^The tumor delivery efficacy of monocyte hitchhiking of liposomal quantum dots relative to free quantum dots.

^c^The tumor delivery efficacy of T cell hitchhiking of DC@AIEdots relative to bare AIEdots.

#### 2.2.1 RBC hitchhiking

RBCs are the most widely used cell type for cell hitchhiking of NMs. The NMs are mainly attached to RBCs ex vivo in a non‐covalent manner. RBC‐hitchhiking technology enables NMs to circulate for a longer period of time in blood and to be delivered to the target organ, for example, the lungs.^[^ ^68^ ^]^ Anselmo et al. demonstrated that RBC hitchhiking significantly extended the circulation lifetimes of the polystyrene (PS) NMs (an approximately 3‐fold increase in blood persistence) and the delivery efficacy to lungs increased about 7‐fold as compared with free NMs.^[^ ^69^ ^]^ The high accumulation of RBC‐bound NMs in the lungs is likely caused by the high shear stress during their passage through the narrow lung capillaries, which means the NMs are transferred to the lung endothelium.^[^ ^70^ ^]^ This natural tissue‐specific targeting capability of RBC hitchhiking has been explored to transport NMs for treating various lung diseases.^[^ ^71^ ^]^ For example, Zhao et al. showed that, compared to free NMs, the RBC‐hitchhiking platform, consisting of DOX‐loaded biodegradable PLGA NMs assembled onto RBCs, achieved 16.6‐fold higher delivery to metastatic lungs 20 min after administration.^[^ ^72^ ^]^ This platform substantially inhibited the progression of lung metastasis and significantly improved the survival in both of the early and the late stages of B16F10‐Luc melanoma lung metastasis models, with expansion of the median survival time by 32 and 8.5 days respectively.

**FIGURE 4:**
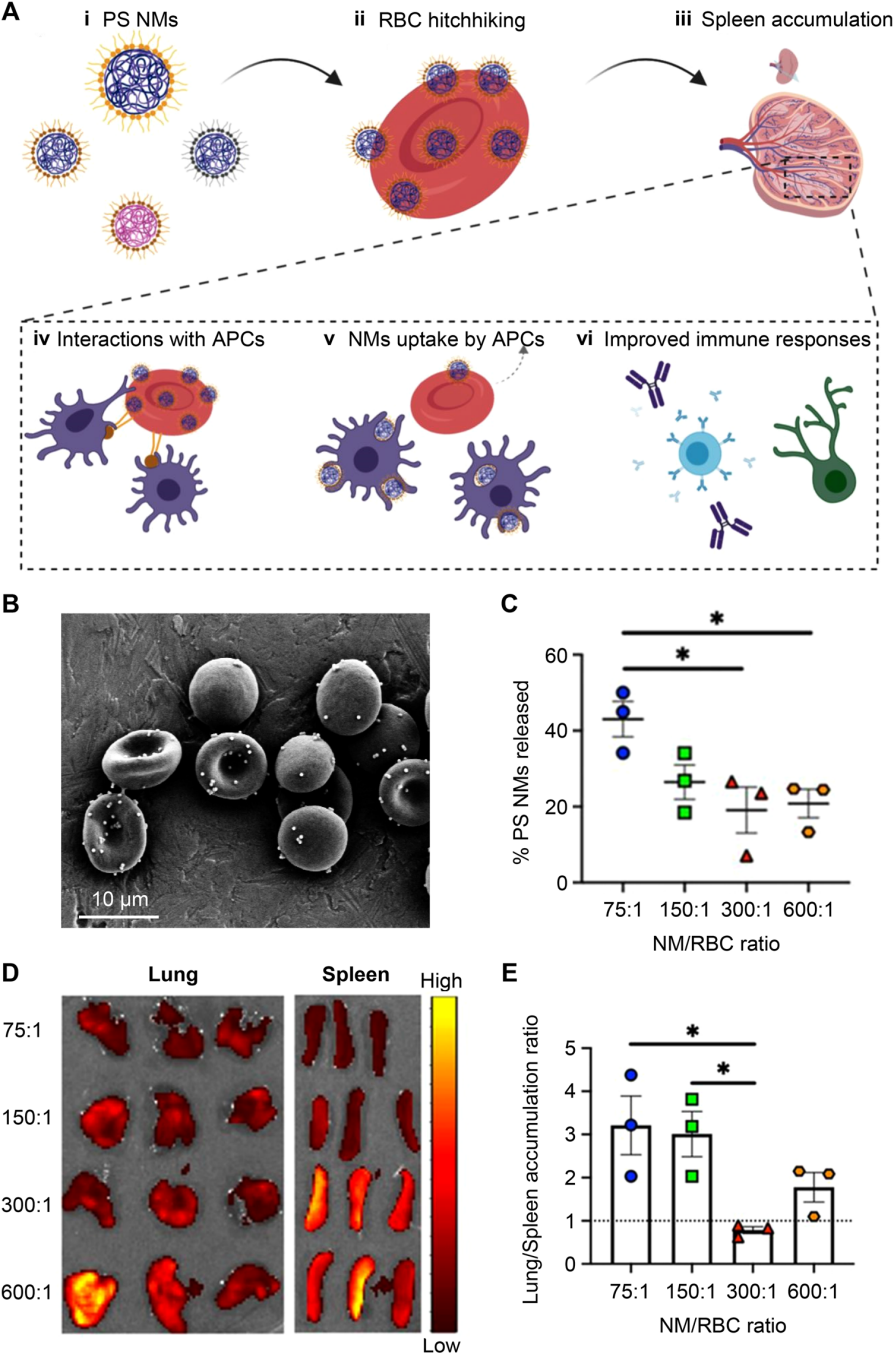
RBC‐hitchhiking system for delivering PS NMs to the spleen. (A) Schematic for the design of delivering PS NMs to the spleen through RBC hitchhiking. (B) SEM of PS NMs‐attached RBCs. (C) Release of PS NMs from RBCs following in vitro shear studies at a shear stress of 6 Pa, corresponding to the shear stress at lung capillaries. (D) In vivo imaging system images of the lungs and spleen collected 20 min after the administration of PS NMs–RBC hitchhiking at different ratios of NMs to RBC. Alexa Fluor 647‐labeled antigen‐coated NMs were used in this experiment. (E) Lung/spleen accumulation ratios calculated by using the fluorescence intensities of isolated lungs and spleen at different ratios of NM/RBC. Reproduced with permission.^[^ ^73^ ^]^ Copyright 2020, National Academy of Sciences.

In addition to lung targeting, Ukidve et al. found that the RBC‐hitchhiking platform predominantly delivered PS NMs to the spleen by enhancing the feed ratios of NMs to RBCs (300:1) (Figure 4).^[^ ^73^ ^]^ This is mainly due to the improved shear resistance of the platform at high NMs loading, which reduces the mechanical dislodgement of the NMs in the lungs and induces their delivery to spleen (Figure 4C–E). Moreover, the authors showed that NMs were delivered to the antigen‐presenting cells instead of macrophages, which have been exploited for immunomodulation.

#### 2.2.2 Neutrophil hitchhiking

Neutrophils represent the first line of host defense against invading foreign agents or microbes. The neutrophils can evade the MPS, cross the BBB, actively home to the sites of tissue damage, infection, or inflammation, and release payloads in response to the highly concentrated inflammatory signals.^[^ ^74^ ^]^ Due to these merits, neutrophils have been employed to deliver many types of NMs including liposome, micelle, MSNs, and polymer to the target tissues.^[^ ^67b^ ^]^ The NMs can be loaded inside the cells via ex vivo incubation or in situ recognition and internalization by circulating neutrophils.^[^ ^75^ ^]^ In one application, Xue et al. harnessed neutrophils to deliver paclitaxel (PTX)‐loaded liposomes (ex vivo hitchhiking) to the inflamed brain tumor of postsurgical glioma‐bearing mice.^[^ ^76^ ^]^ The neutrophils hitchhiking treatment efficiently slowed the onset of malignant glioma recurrence, extending the survival time by 50%, from 29 to 61 days. Wu et al. developed a neutrophil hitchhiking platform by in vitro assembling of magnetic MSNs into neutrophils. The active targeting of NMs‐loaded neutrophils toward the inflamed glioma sites was then successfully tracked by magnetic resonance imaging.^[^ ^77^ ^]^

Owing to the short lives of neutrophils (approximately 7 h) and the risk of contamination by ex vivo hitchhiking, the approach of in situ hitchhiking neutrophils has drawn much attention in recent years. For example, cRGD‐modified liposomes were shown to be preferentially internalized by circulating neutrophils/monocytes via cRGD and integrin α_v_β_1_ receptor. This cell carrier system effectively delivered the therapeutic molecules to the brain lesion sites and achieved enhanced protection against ischemic stroke in a cerebral ischemia (CI)/reperfusion rat model.^[^ ^78^ ^]^ Inspired by the fact that neutrophils in nature combat invading pathogens such as bacteria and viruses, Li et al. developed a nanopathogenoid (NPN) system by coating drug‐loaded micelle with bacterial outer membrane vesicles for tumor‐targeted delivery.^[^ ^79^ ^]^ This NPN system hitchhiked onto blood neutrophils in situ and then actively homed into tumors, where they were released from neutrophils in response to inflammatory signals. Furthermore, they found that the inflammatory microenvironment created by PTT in tumors further improved the accumulation of NPN‐loaded neutrophils in tumors as compared to the untreated controls. The combination treatment of cisplatin‐loaded NPNs and PTT completely eradicated the tumors in all treated mice.

#### 2.2.3 Monocyte/macrophage‐hitchhiking

In addition to neutrophils, other types of leukocytes, including monocytes and macrophages, have also been actively used as living cell carriers.^[^ ^80^ ^]^ Like neutrophils, both monocytes and macrophages can escape from the MPS sequestration, across the BBB, and have excellent inflammatory tropism, while they do not release payloads under) inflammatory conditions.^[^ ^81^ ^]^ Monocytes/macrophages have excellent active tumor targeting abilities, and can be recruited into the hypoxic areas of tumors owing to the elevated expression of chemokines.^[^ ^82^ ^]^ Additionally, the circulation times of these cells (∼20 days) are longer than that of neutrophils (∼7 h). NMs are mainly internalized or attached to the surface of macrophages ex vivo. Gao et al. developed a cucurbit[7]uril‐based supramolecular macrophage‐liposome (M‐L) marriage for the delivery of drug‐loaded liposomes to inflammatory tissues, including melanoma and acute pneumonia.^[^ ^83^ ^]^ Peritoneal macrophages were modified with cucurbit[7]uril via lipid ligand membrane insertion and incubated ex vivo with adamantane‐liposomes to obtain the M‐L marriage. Negligible effects on the migratory and invasive abilities of the cells were observed. This supramolecular macrophage hitchhiking significantly enhanced the delivery of drug‐loaded liposomes to the inflammatory tissues and achieved a better therapeutic effect than free liposomes.

Monocytes are blood‐borne precursor of tissue macrophages and DCs. Similar to macrophages, they can target tumors and have natural phagocytic abilities.^[^ ^84^ ^]^ For monocyte hitchhiking, the advanced approach to load NMs is in situ internalization by circulating cells or in situ attachment to the cell surface via specific ligand‐receptor interactions.^[^ ^85^ ^]^ Taking advantage of the natural phagocytic behavior of monocytes/macrophages to take up cell debris, Zheng et al. used a NMs‐loaded apoptotic body (AB) as the vector for intratumor‐targeted delivery. The gold‐silver nanorod‐loaded ABs (AuNR/ABs) were selectively engulfed by circulating Ly‐6C^+^ monocytes. Based on flow cytometry result, more than 80% of monocytes phagocytosed the AuNR/ABs. Hitchhiking on monocytes enabled AuNR/ABs to effectively evade MPS clearance and actively infiltrate into the inner region of tumor via their prominent tumor‐homing tendency.^[^ ^86^ ^]^

However, because the internalization of NMs by monocytes may result in their lysosomal degradation, efforts have been made to attach NMs to the surface of cell instead. To specifically target circulating monocytes, Huang et al. selected a specific monocyte‐targeting aptamer through the cell systematic evolution of ligands by exponential enrichment (Cell‐SELEX) and integrated it with lipid nanoparticles (LNPs) to produce a monocyte‐targeting delivery platform.^[^ ^87^ ^]^ The drug‐loaded platform was capable of attaching selectively onto circulating monocytes surface, which efficiently delivered the drugs (IOX2 or gemcitabine) to the diseased sites, including myocardial ischaemia‐reperfusion (IR) injury and pancreatic cancer. This platform not only successfully ameliorated IR injury but inhibited tumor growth in a pancreatic cancer mouse model, showing significant improvements in survival and healing.

#### 2.2.4 Lymphocyte hitchhiking

Lymphocytes, particularly T cells, have also been exploited as living carriers.^[^ ^88^ ^]^ Lymphocytes possess potent immunological functions, such as cell‐mediated cytotoxicity.^[^ ^89^ ^]^ Additionally, they can evade the MPS sequestration, improve circulation, and target tumors or lymph nodes.^[^ ^90^ ^]^ Xu et al. recently developed DC membrane‐coated aggregation‐induced emission photosensitizer (DC@AIEdot) with hitchhiking and antigen‐presenting abilities for cancer photodynamic immunotherapy.^[^ ^91^ ^]^ The DC@AIEdot was capable of hitchhiking onto the endogenous T cells via the interactions between the ligands on DC membranes (MHC, CD80/86, etc.) and receptors on T cells, and achieved a 1.6‐fold increase in tumor delivery compared with the bare AIEdot. Furthermore, the DC membranes of DC@AIEdot were able to activate T cells in vivo and trigger an antitumor response. The combination of immunotherapy with lipid droplet‐targeted PDT of DC@AIEdot effectively repressed the growth of primary and distant tumors. In addition to DC membrane‐coating, E‐selectin modification has also been reported as an effective way to adhere NMs to the surface of circulating leukocytes for improved drug delivery to tumors.^[^ ^92^ ^]^

#### 2.2.5 Stem cell hitchhiking

Stem cells with the intrinsic tropism to seek specific diseased/pathological sites, such as tumor, inflammation, and injury, have emerged as an attractive cell type for hitchhiking of NMs.[^59b^ ^]^ For instance, mesenchymal stem cells (MSCs) can be relatively easily isolated from the bone marrow of patients and are a good candidate for delivering NMs to the target sites.^[^ ^93^ ^]^ MSC carriers have been used to transport many types of NMs to tumors,^[^ ^94^ ^]^ e.g., delivering photosensitizer‐loaded MSNs to breast tumors,^[^ ^95^ ^]^ transporting chlorin e6‐conjugated polydopamine NMs to lung metastatic sites, etc.^[^ ^96^ ^]^

Another promising carrier is neural stem cells (NSCs) with innate tumor tropism. They show promise on drug delivery to brain tumors, although the isolation of autologous NSCs from neurogenic areas in the brain remains a major challenge.^[^ ^97^ ^]^ Such a challenge can be overcome by reprogramming patients’ own somatic cells to produce engineered NSCs.^[^ ^98^ ^]^ For example, the second‐generation of induced NSCs was recently developed by Jiang et al. by direct transdifferentiation of human fibroblasts using a SOX2 single‐factor cell reprogramming method.^[^ ^99^ ^]^ These induced NSCs showed superior abilities to migrate to human triple‐negative breast cancer (TNBC) cells and to home to TNBC brain parenchymal tumors following intracerebroventricular infusion.

Overall, this section demonstrates the versatility of cell hitchhiking approach for protecting NMs from MPS clearance and delivering them to target tissues. This relies mainly on the natural function of different cells, such as immune evasion and intrinsic tropism. However, it is worth noticing that NMs loading may impair these primary functions. For example, while RBC hitchhiking possesses natural lung‐targeting ability, high NMs attachment to the cells induces a high spleen accumulation instead of lungs.^[^ ^73^ ^]^ Though MSCs have intrinsic tropism to tumors, high AuNPs loading inside the cells leads to high accumulation in the lungs.^[^ ^100^ ^]^ Future investigations should be undertaken not only to develop new methods to efficiently hitchhike target cells and preserve the physicochemical properties of NMs, but also carefully evaluate the effects of NMs loading on the primary function of cells.

### 2.3 Physiological environment modulation

**FIGURE 5:**
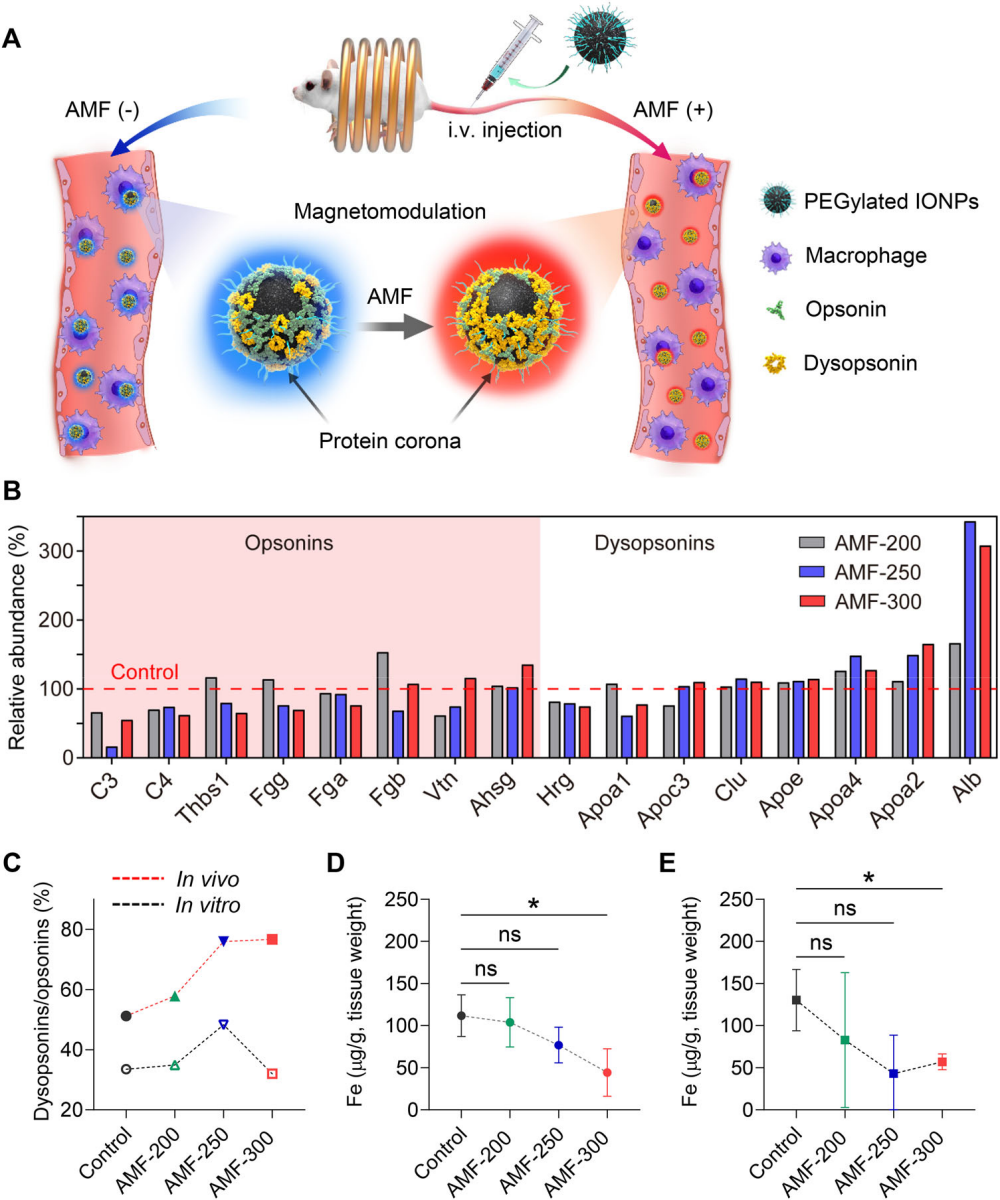
Magnetothermal manipulating the corona composition of circulated iron oxide nanoparticles (IONPs) and changing their distribution in the body. (A) Schematic diagram depicting magnetothermal regulation of the corona on circulated IONPs, wherein in situ corona regulation leads to downregulated opsonins and upregulated dysopsonins, which further decreases sequestration by the MPS. (B) The impact of in vivo magnetothermal regulation on the relative abundance of opsonins and dysopsonins (top 20 most abundant) on circulated IONPs. The IONPs without AMF treatment served as control. (C) Variation of the dysopsonin‐to‐opsonin ratios after magnetothermal regulation both in vitro and in vivo. Liver (D) and spleen (E) distribution of IONPs 12 h post‐injection with different treatments. AMF conditions: frequency, *f* = 345 kHz and magnetic field amplitude, *H* = 200, 250, and 300 Oe (AMF‐200, AMF‐250, and AMF‐300); duration, 15 min. Reproduced with permission.^[^ ^104^ ^]^ Copyright 2021, Elsevier.

In addition to the physical and chemical properties of NMs, the physiological environment also has a significant impact on MPS sequestration of NMs via manipulating their corona components.^[^ ^101^ ^]^ For example, the incubation temperature has been demonstrated to influence protein‐NM interactions and to lead to the variations of corona components on NMs.^[^ ^102^ ^]^ Furthermore, Suslick et al. showed that laser‐induced local heating significantly changed the corona components on gold nanorods (GNRs), and local heating was more efficient than bulk heating.^[^ ^103^ ^]^ However, concerns about phototoxicity and the limited penetration depth of photons hindered its in vivo application. To manipulate the corona composition of circulated IONPs in vivo, Zhang et al. developed a magnetothermal regulation approach that relied on localized heat generated by IONPs in response to a safe and deeply penetrating alternating magnetic field (AMF) (Figure 5A).^[^ ^104^ ^]^ Upon in vivo magnetothermal regulation, the corona on IONPs showed downregulated opsonins and upregulated dysopsonins, and the dysopsonin‐to‐opsonin ratio increased with increasing magnetic field amplitude (*H*) (Figure 5B,C). The variations in corona components on IONPs contributed to the decrease in MPS sequestration, resulting in reduced accumulation in the livers (60.4% decrease) and spleens (56.3% decrease) of healthy mice (Figure 5D,E). The magnetothermal regulation approach provides a new possibility to alter the in vivo fate of IONP‐based nanosystems in situ toward improved cancer nanotherapy. In addition to temperature, other physiological environment factors such as serum concentration and dynamic conditioning environment have been well reviewed in recent studies.^[^ ^8a^ ^]^

## 3 DISABLING MPS TO MINIMIZE NMs CLEARANCE

In addition to engineering NMs for MPS evasion, the approach that directly disables the MPS function has also been actively applied to minimize the sequestration of NMs by MPS.^[^ ^105^ ^]^ One method is to temporarily block the MPS using decoy NMs or cells. Another method is to transiently inhibit the phagocytic activity of macrophages using pharmacological endocytosis inhibitors. In the latter method, macrophages are directly depleted using clodronate, zoledronate, and so forth.

### 3.1 MPS blockade

MPS blockade is an approach that temporarily saturates macrophages using decoy agents, thereby facilitating the evasion of MPS clearance of the subsequently administered NMs. The typical method to block the MPS is pre‐treatment with a large dose of low‐toxic “blocking” NMs to occupy macrophages, such as liposomes,^[^ ^106^ ^]^ polymer NPs,^[^ ^107^ ^]^ MSNs, etc.^[^ ^108^ ^]^ The efficacy of MPS blockade using this approach is demonstrated to be closely related to the physicochemical properties of the blocker, including size, dose, and surface chemistry. For example, to decrease the sequestration by Kupffer cells (KC) in the liver, Saunders et al. administered an optimized liposome with a hydrodynamic diameter of > 230 nm prior to the LNPs formulation containing siRNA or mRNA.^[^ ^109^ ^]^ It was found that the liposome blocker was more inclined to be internalized by KC than hepatocytes, probably due to their larger size than that of the fenestrae of liver capillaries, thus hindering the liposome from interacting with hepatocytes. Furthermore, the blood bioavailability of LNPs was closely related to the blocker dosage, where a higher dose induced a greater improvement. Pre‐treatment with a blocker at 360 mg/kg resulted in 49% and 32% improvements in the gene silencing and expression efficacies of LNPs, as compared with LNPs treatment alone. To reduce the dose of the blocker, Tang et al. developed a “Do not eat me” blocker, characterized by a CD47‐derived “self” peptide‐modified liposome that can reside on macrophage membranes over a long period.^[^ ^110^ ^]^ As a result, a much lower dose (100 mg/kg) of “self” liposomes significantly prolonged the circulation time of the subsequently injected NMs as compared with conventional liposomes (400 mg/kg). This approach has also been demonstrated to be useful in different formulations such as liposome‐based nanomedicines,^[^ ^111^ ^]^ GNRs^[^ ^112^ ^]^ and IONPs,^[^ ^113^ ^]^ demonstrating the generalizability of the approach to evade the MPS clearance and improve the bioavailability of NMs. As macrophages of the MPS play a pivotal role in innate immune system and involve in numerous biological processes, future studies should investigate the potential toxicity caused by the interactions between the blocker and the MPS.^[^ ^114^ ^]^

**FIGURE 6:**
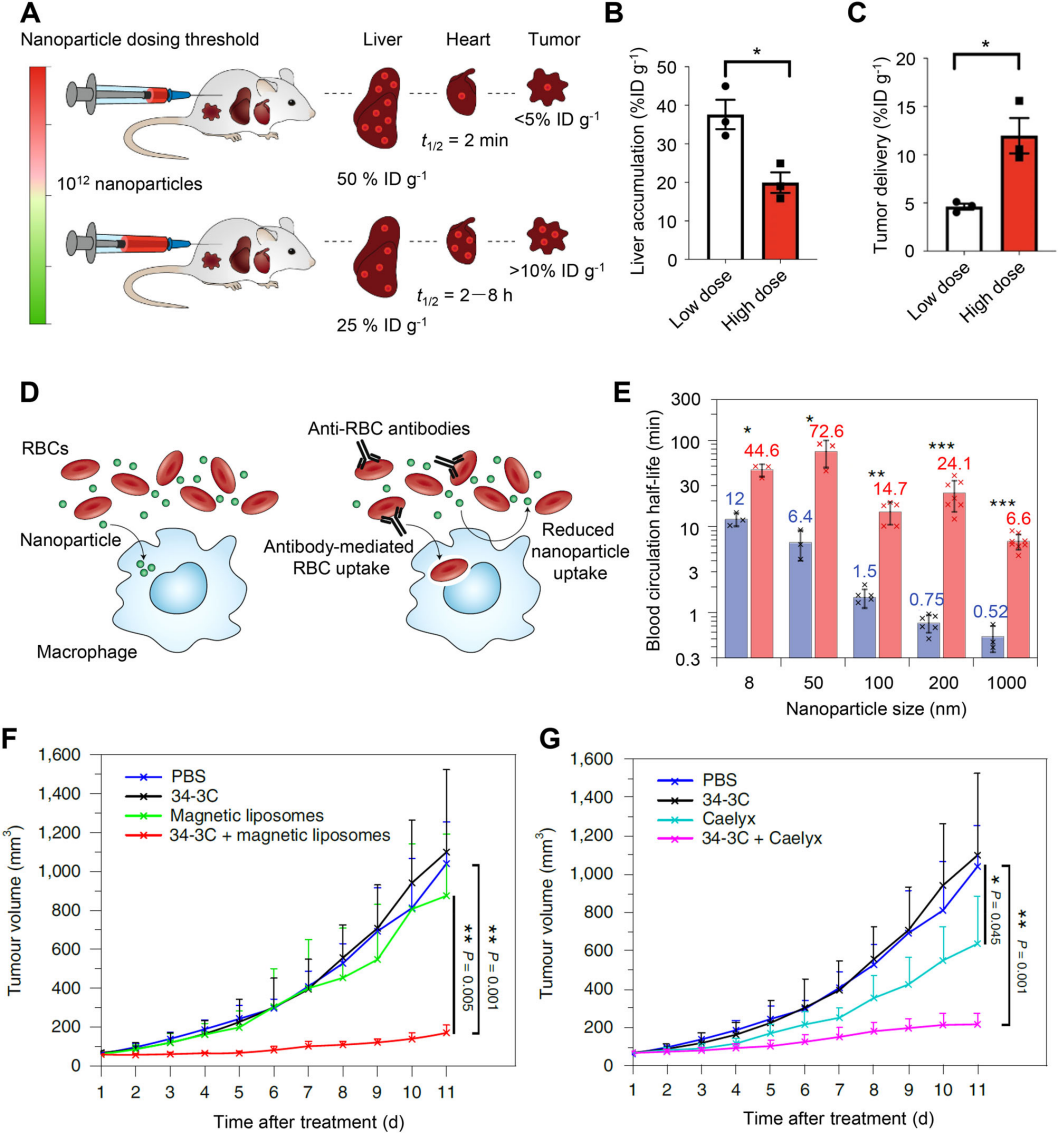
MPS blockade approaches to reduce the MPS clearance and improve tumor delivery efficacy of NMs. (A) The injection dose of NMs determines their distribution and tumor delivery. Reproduced with permission.^[^ ^117^ ^]^ Copyright 2020, Springer Nature. High doses of AuNPs reduce the accumulation in the liver (B) and increase tumor delivery (C) in xenogeneic U87 glioma‐bearing mice. Reproduced with permission.^[^ ^115^ ^]^ Copyright 2020, Springer Nature. (D) Anti‐erythrocyte‐mediated erythrocytes clearance blocks the MPS and enhances the bioavailability of intravenously injected NMs. Reproduced with permission.^[^ ^118^ ^]^ Copyright 2020, Springer Nature. (E) The circulation half‐lives of both short‐ and long‐circulating NMs with (test group, red) and without (control group, blue) MPS cytoblockade. Short‐circulating NMs: 200‐nm Estapor (Merck Millipore), 1‐μm Dynabeads MyOne (Thermo Fisher Scientific); long‐circulating NMs: 8 nm CdSe/ZnS quantum dots, 50‐ and 100‐nm fluidMAG‐ARA (Chemicell). Plot of tumor volume versus time after the treatment of doxorubicin‐loaded magnetic liposomes (F) or Caelyx (G) with and without 34‐3C‐induced MPS‐cytoblockade in B16‐F1 melanoma‐bearing mice. Reproduced with permission.^[^ ^116^ ^]^ Copyright 2020, Springer Nature.

Ouyang et al. recently demonstrated a dose‐dependent threshold principle for reducing the sequestration by liver KC and improving NMs tumor delivery efficacy (Figure 6A).^[^ ^115^ ^]^ In mice, the threshold was 1 trillion NMs, and NMs doses above the threshold can overwhelm the KC uptake rates, resulting in decreased liver clearance and increased tumor delivery (Figure 6B,C). The maximum tumor delivery efficiency of AuNPs at doses beyond the threshold was up to 12% of the injected dose. Furthermore, the dose threshold principle was generally true for different NMs compositions (silica and liposome) and was demonstrated to be more powerful than the conventional MPS blockade approach in improving the pharmacokinetics and tumor delivery of NMs. Toward future clinical translation, the dose‐related toxicity of this approach needs to be carefully evaluated in a wide range of animal models.

Nikitin et al. proposed a new method named MPS cytoblockade, which is different from the MPS blockade approach using large doses of decoy agents (Figure 6D).^[^ ^116^ ^]^ This method used organisms’ own erythrocytes as a blocker to induce MPS saturation and thus decrease the phagocytic elimination of circulated NMs. In a healthy mouse model, the authors administered a mouse monoclonal antimouse‐RBC antibody (IgG2a 34‐3C, 1.25 mg/kg) to induce erythrophagocytosis and then injected NMs. The short‐ and long‐circulating NMs were both lengthened up to 32‐fold, as compared with the group without MPS cytoblockade (Figure 6E). In addition, MPS cytoblockade has also succeeded in improving the solid tumor targeting of non‐stealth fluidMAG‐ARA particles (Chemicell) and enhancing tumor treatment with both magnetic liposomes (Figure 6F) and clinically approved Caelyx (Figure 6G).

### 3.2 Suppression of macrophage phagocytosis

**FIGURE 7:**
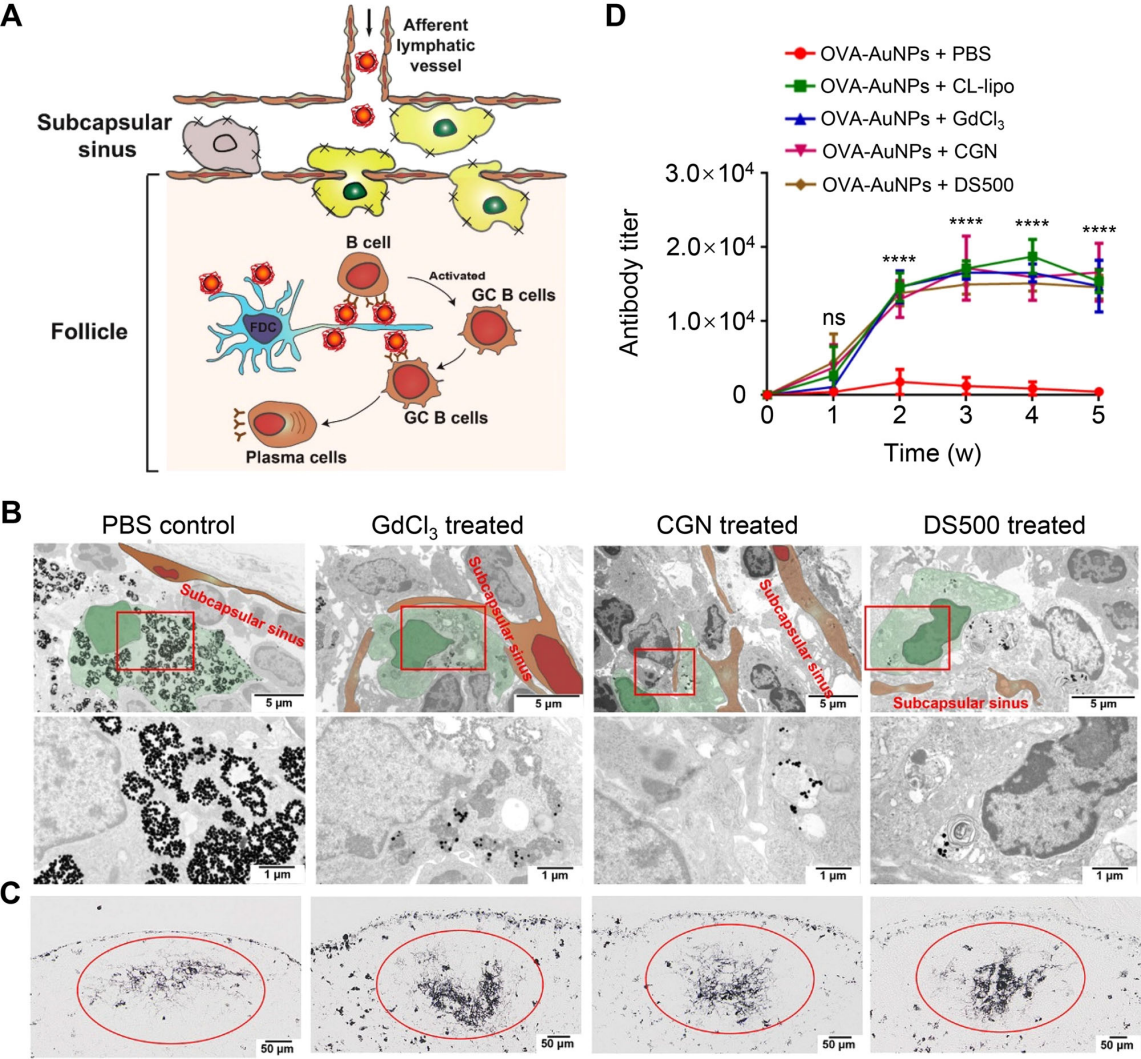
Suppressing SCS macrophages improves the delivery of nanovaccines to lymph node follicles for robust humoral immunity. (A) Scheme for improving the delivery of OVA‐AuNPs nanovaccines to follicular dendritic cells by suppression of SCS macrophage phagocytosis for robust humoral immune responses. Germinal center B cells (GC B cells). (B) Representative TEM images and the magnified TEM images of SCS macrophages after pre‐treatment of macrophage inhibitors for 24 h and intradermal footpad administration of OVA‐AuNPs nanovaccines. SCS macrophages are shown in green, and lymphatic endothelial cells are shown in brown. (C) Histological images showing the accumulation of OVA‐AuNPs nanovaccines in follicles 48 h after intradermal footpad administration. The mice were pre‐treated with macrophage inhibitors for 24 h prior to nanovaccine injection. (D) Production of OVA‐specific antibodies after injection of macrophage inhibitors and OVA‐AuNP nanovaccines. CL‐lipo, clodronate liposomes, are able to remove macrophages. Reproduced with permission.^[^ ^124^ ^]^ Copyright 2020, American Chemical Society.

Another approach developed by researchers to transiently diminish the endocytosis of NMs by MPS is by using pharmacological endocytosis inhibitors.^[^ ^119^ ^]^ Chloroquine is a clinically approved malaria drug and has been used as a broad‐spectrum endocytosis inhibitor.^[^ ^120^ ^]^ Wolfram et al. recently demonstrated that pre‐treatment with chloroquine effectively reduced the sequestration of liposomes by the MPS, resulting in reduced liver accumulation (64% decrease) and enhanced tumor delivery (2‐fold improvement), as compared with the control. Mechanistic studies showed that chloroquine treatment induced lysosomal dysfunction by preventing lysosome acidification and reduced the expression of phosphatidylinositol‐binding clathrin assembly protein, which plays a key role in clathrin‐dependent nanoparticle uptake. In addition to tumor delivery, the resident macrophages in the subcapsular sinus (SCS), are also a barrier to nanovaccine delivery into lymph node follicles. It was shown by Zhang et al. that treatment of macrophage endocytosis inhibitors, such as gadolinium chloride (GdCl_3_), dextran sulfate 500 (DS500), and carrageenan (CGN), significantly reduced the sequestration of ovalbumin‐conjugated AuNPs (OVA‐AuNPs) nanovaccines by SCS macrophages, and increased the accumulation of nanovaccines in follicles by 2‐fold, compared to the PBS group (Figure 7A–C).^[^ ^121^ ^]^ Coadministration of 0.1‐mg macrophage inhibitors and OVA‐AuNPs induced greater humoral immune responses and resulted in > 30‐fold higher OVA‐specific antibody production than nanovaccines alone in 5 weeks, suggesting that the macrophage inhibitors can serve as adjuvants to be formulated with nanovaccines to boost humoral immunity (Figure 7D). In addition, esomeprazole,^[^ ^122^ ^]^ a clinically approved proton‐pump inhibitor, and methyl palmitate,^[^ ^123^ ^]^ a natural fatty acid, have also been demonstrated to be effective in reducing NMs uptake by the MPS.

### 3.3 Depletion of macrophages

A more aggressive approach to inhibit the sequestration of NMs by the MPS is transient depletion of macrophages in the liver or spleen by using clodronate,^[^ ^125^ ^]^ zoledronate,^[^ ^114^ ^]^ and so forth. Although macrophages remove most administered NMs from the bloodstream, they also play a key role in the innate immune system. Therefore, depletion of the macrophages may weaken the host innate immune system and impair their ability to combat infection. To minimize the potential toxicity of macrophage deletion, researchers have developed macrophage‐specific formulation such as clodronate liposome to only deplete a portion of macrophages by controlling the drug dosage. Tavares et al. screened the optimal clodronate liposome dose needed to deplete macrophages and evaluated the effect of macrophage depletion on the NMs delivery to tumors.^[^ ^126^ ^]^ Their results showed that administration of 17‐ and 50‐mg/kg clodronate liposomes decreased the KC to 25% and 10%, respectively, as compared with the control group. Although high doses of clodronate liposomes induced lower accumulation of AuNPs in the liver, their tumor delivery efficacy improvement was not statistically significant, indicating that complete removal of macrophages did not maximize nanoparticle tumor delivery. For 100‐nm AuNPs, pre‐treatment with a low dose of clodronate liposome induced a 100‐fold enhancement in tumor delivery in PC3 tumor‐bearing mice. Moreover, this approach can also enhance the transport of nanoliposomes (by 2‐fold), silica nanoparticles (by 5‐fold), and silver nanoparticles (by 7‐fold) to tumor sites in SKOV3 tumor‐bearing mice, demonstrating the universality of this approach to enhance the tumor delivery of NMs. Ackun‐Farmmer et al. demonstrated that pre‐treatment with clodronate liposome led to a 3‐ and 1.8‐fold reduction of bone‐targeted NPs in the spleen and liver, respectively, resulting in a significant enhancement in bone accumulation.^[^ ^127^ ^]^

To enhance nanodrug delivery to pulmonary fibrotic tissues, Sun et al. developed a clodronate liposomal and fibroblast‐derived exosomal hybrid drug nanocarrier, where the clodronate can deplete the KC via passive targeting and the homing properties of homologous exosome favor pulmonary fibrosis‐specific accumulation and penetration.^[^ ^128^ ^]^ The nanocarrier significantly enhanced the delivery of nintedanib, an antifibrotic agent approved for treating pulmonary fibrosis, to pulmonary fibrotic lesions and achieved a remarkable improvement in treating pulmonary fibrosis. This treatment notably prolonged the survival of pulmonary fibrosis mice from 12.5% to 87.5% in 60 days. The low dose of clodronate (15 mg/kg) induced no obvious damage to liver function and did not interfere with the body's innate immunity.

## 4 CONCLUSIONS AND PERSPECTIVES

In this review, we introduced recent advances in strategies to evade the MPS clearance of NMs, with the aim of improving the accumulation of NMs in targeted disease sites. We summarized and divided these advances into two categories. The first strategy is to engineer NMs to minimize the recognition and sequestration of NMs by the MPS. The second strategy is to manipulate the MPS function to suppress their NMs elimination capability. These advances will lay a good foundation for the development of NMs with a high MPS‐evading ability and this will contribute NMs to achieve better therapeutic effects.

Current studies on evading the MPS clearance of NMs focus mainly on the first strategy (Engineering NMs). In particular, cell membrane‐coating and cell hitchhiking are emerging and highly promising approaches for biomimetic functionalization. These approaches not only mask immune recognition and prolong circulation but also endow NMs with additional functionalities such as intrinsic tropism to target tissues. Many types of cells, such as RBCs, leukocytes, and stem cells, as well as different types of NMs, such as polymers and inorganic NMs, have been applied by these approaches for drug delivery, phototherapies, or immunotherapies. However, several critical barriers need to be addressed to enable the future development and clinical translation of these approaches. For example, the cell membrane coating‐approach requires the development of high yielding and standardized methods for membrane derivation from anucleate or nucleate cells. In addition, the long‐term biological safety of different components and types of cell membranes needs to be determined. For the cell hitchhiking approach, a key challenge is the development of specific and efficient methods to facilitate NMs to hitch a ride on target circulating cells in situ. These methods offer many advantages, including the circumvention of labor‐intensive cell isolation and preloading processes and the alleviation of immunogenicity risk. Meanwhile, the adverse effects of NMs hitchhiking on the normal activity or primary functionality of cells should be carefully investigated. Furthermore, cell hitchhiking requires a better understanding of the biology characteristics of target cells.

Our review suggests that the second strategy (Disabling MPS) has received relatively less attention compared to the first strategy. However, recent advances associated with the second strategy have clearly demonstrated its effectiveness. Nevertheless, further efforts are needed for development of new, safe, and effective methods to disable the MPS. For instance, one attractive goal is to develop a universal and highly efficient blocker that can circumvent the dose‐related toxicity associated with the conventional MPS blockade approach. The macrophage phagocytosis suppression and macrophage depletion approaches require developing macrophage‐specific delivery platforms to reduce the adverse effects on non‐macrophages. Macrophage‐targeting aptamers or peptide‐modified nanocarriers are ideal platforms as they can selectively attach onto the surface of macrophages.^[^ ^129^ ^]^ Furthermore, biomimetic nanocarriers, such as apoptotic bodies and pathogen‐based vectors, that can be selectively phagocytosed by macrophages,^[^ ^130^ ^]^ are favorable candidates. The type and dose of endocytosis inhibitors or drugs should be also optimized to achieve precise control of MPS function while minimizing unwanted effects on the innate immune system.

Combining both strategies may also provide a way to overcome the MPS barrier. This approach mainly relies on NMs that should be rationally designed to simultaneously prevent MPS interactions and disable the MPS. However, incorporating multiple functions in the same NMs will also increase their complexity, leading to poor scalability and reproducibility, thus hindering their clinical translation. Therefore, if the goal is clinical translation, the development of simple but effective NMs may be the best approach.

## CONFLICT OF INTEREST

The authors declare no conflict of interest.
